# Population pharmacokinetics and dose evaluations of linezolid in the treatment of multidrug-resistant tuberculosis

**DOI:** 10.3389/fphar.2022.1032674

**Published:** 2023-01-09

**Authors:** Haoyue Zhang, Yuying He, Lina Davies Forsman, Jakob Paues, Jim Werngren, Katarina Niward, Thomas Schön, Judith Bruchfeld, Jan-Willem Alffenaar, Yi Hu

**Affiliations:** ^1^ Department of Epidemiology, School of Public Health and Key Laboratory of Public Health Safety, Fudan University, Shanghai, China; ^2^ Institute of Tuberculosis Control, Guizhou Provincial Center for Disease Control and Prevention, Guiyang, China; ^3^ Department of Infectious Diseases, Karolinska University Hospital, Stockholm, Sweden; ^4^ Department of Medicine, Division of Infectious Diseases, Karolinska Institute, Stockholm, Sweden; ^5^ Department of Biomedical and Clinical Sciences, Linköping University, Linköping, Sweden; ^6^ Department of Infectious Diseases, Linköping University Hospital, Linköping, Sweden; ^7^ Department of Microbiology, The Public Health Agency of Sweden, Stockholm, Sweden; ^8^ Department of Infectious Diseases, Kalmar County Hospital, Linköping University, Kalmar, Sweden; ^9^ University of Sydney, Faculty of Medicine and Health, School of Pharmacy, Sydney, NSW, Australia; ^10^ Westmead Hospital, Sydney, NSW, Australia; ^11^ Sydney Institute for Infectious Diseases, University of Sydney, Sydney, NSW, Australia

**Keywords:** linezolid, pharmacokinetics, dose evaluation, multidrug-resistant tuberculosis, pharmacodynamics

## Abstract

**Background:** The pharmacokinetic/pharmacodynamics (PK/PD) target derived from the hollow-fiber system model for linezolid for treatment of the multidrug-resistant tuberculosis (MDR-TB) requires clinical validation. Therefore, this study aimed to develop a population PK model for linezolid when administered as part of a standardized treatment regimen, to identify the PK/PD threshold associated with successful treatment outcomes and to evaluate currently recommended linezolid doses.

**Method:** This prospective multi-center cohort study of participants with laboratory-confirmed MDR-TB was conducted in five TB designated hospitals. The population PK model for linezolid was built using nonlinear mixed-effects modeling using data from 168 participants. Boosted classification and regression tree analyses (CART) were used to identify the ratio of 0- to 24-h area under the concentration-time curve (AUC_0-24h_) to the minimal inhibitory concentration (MIC) threshold using the BACTEC MGIT 960 method associated with successful treatment outcome and validated in multivariate analysis using data from a different and prospective cohort of 159 participants with MDR-TB. Furthermore, based on the identified thresholds, the recommended doses were evaluated by the probability of target attainment (PTA) analysis.

**Result:** Linezolid plasma concentrations (1008 samples) from 168 subjects treated with linezolid, were best described by a 2-compartment model with first-order absorption and elimination. An AUC_0–24h_/MIC > 125 was identified as a threshold for successful treatment outcome. Median time to sputum culture conversion between the group with AUC_0-24h_/MIC above and below 125 was 2 *versus* 24 months; adjusted hazard ratio (aHR), 21.7; 95% confidence interval (CI), (6.4, 72.8). The boosted CART-derived threshold and its relevance to the final treatment outcome was comparable to the previously suggested target of AUC_0–24h_/MIC (119) using MGIT MICs in a hollow fiber infection model. Based on the threshold from the present study, at a standard linezolid dose of 600 mg daily, PTA was simulated to achieve 100% at MGIT MICs of ≤ .25 mg which included the majority (81.1%) of isolates in the study.

**Conclusion:** We validated an AUC_0–24h_/MIC threshold which may serve as a target for dose adjustment to improve efficacy of linezolid in a bedaquiline-containing treatment. Linezolid exposures with the WHO-recommended dose (600 mg daily) was sufficient for all the *M. tb* isolates with MIC ≤ .25 mg/L.

## 1 Introduction

With the update of World Health Organization (WHO) multidrug-resistant tuberculosis (MDR-TB) treatment guidelines in 2019, linezolid is included as one of the important Group A agent (World Health, 2020). It is expected that this will improve the 6-month sputum culture conversion rate as well as treatment outcome of MDR-TB treatment (Lee et al., 2017; Ahmad et al., 2018; Singh et al., 2019; Padayatchi et al., 2020).

Linezolid is a drug with a narrow therapeutic window which requires close monitoring of participants to prevent toxicity (Wasserman et al., 2016). In the MDR-TB treatment recommendations issued in 2020 (World Health, 2020), the WHO highlighted the urgent need to investigate linezolid dose optimization and treatment duration in order to minimize its toxicity. As linezolid drug concentrations are highly variable (Wasserman et al., 2019), therapeutic drug monitoring (TDM) is recommended to monitor the drug exposure to facilitate dose individualization. Previous studies have consistently shown that higher drug exposure of linezolid in relation to *in vitro* susceptibility of the *Mycobacterium tuberculosis (M. tb)* isolate (Heinrichs et al., 2019) is associated with improved TB treatment outcome (Pasipanodya et al., 2013; Swaminathan et al., 2016; Zheng et al., 2021).

The ratio of 0- to 24-h area under the concentration-time curve (AUC_0-24h_) to the minimal inhibitory concentration (MIC) is generally used as the thresholds in TDM of TB treatment (Sturkenboom et al., 2021). A study in a hollow fiber infection model of tuberculosis found that optimal microbial kill for linezolid was achieved at an AUC_0-24h_/MIC ratio of 119 (Srivastava et al., 2017). However, this target was identified *in vitro*, using linezolid in monotherapy and only a single *M. tb* strain H37Rv (ATCC 27294) with MIC identified using the mycobacterial growth indicator tube (MGIT) assay (Becton Dickinson, Franklin Lakes, NJ) (Srivastava et al., 2017). Thus, it is necessary to validate the clinical relevance of previously reported target and to evaluate the sufficiency of the recommended and commonly used doses of linezolid. Therefore, the present study aimed to model the population pharmacokinetics (PK) of linezolid when administered as part of a standardized MDR-TB treatment regimen, identify pharmacokinetic/pharmacodynamics (PK/PD) threshold associated with treatment outcome and to evaluate the current dose of linezolid.

## 2 Method

### 2.1 Study design

Participants from two cohorts were included for this analysis. The development cohort derived from a previously reported study (Zheng et al., 2021), targeting the participants with bacteriological diagnosis of MDR-TB from designated hospitals in Jiangsu, Guizhou and Sichuan Province in China between January 2015 and December 2017. The validation cohort enrolled participants from Sichuan, Jiangsu and Henan Province based on the same inclusion criteria. Briefly, eligible participants were ≥ 18 years old and < 70 years old and diagnosed as MDR-TB by GeneXpert MTB/RIF (Cepheid, Sunnyvale, CA) and *M. tb* drug susceptible test. Participants were excluded if critically ill, pregnant, infected with HIV, HBV or HCV, having received treatment for MDR-TB for more than 1 day, or refused to participate.

The development cohort received linezolid-containing regimen including bedaquiline, moxifloxacin or levofloxacin, linezolid as well as the background regimen to complete a full-oral regimen. The validation cohort received a standardized oral regimen of fluoroquinolones, bedaquiline, linezolid, clofazimine and cycloserine for 6 months, followed by fluoroquinolones, linezolid, clofazimine and cycloserine for 18 months (World Health, 2020). All participants were given 600 mg linezolid once daily, as recommended by the WHO (World Health, 2020). The study was approved by the ethics committee of the School of Public Health, Fudan University (IRB#2015-08-0565) and written informed consent was obtained from all subjects.

In both two study cohorts, the participant received the inpatient treatment for the first 2 weeks after treatment initiation, then followed by outpatient treatment. All study participants were routinely examined monthly during the intensive phase (the first 6 months) and once every 2 months during the consolidation phase (the next 18 months). A questionnaire was used to collect demographic data, while medical and laboratory data were extracted from hospital records.

### 2.2 Blood drug concentration determination

In the development cohort, blood samples for drug concentration analysis were collected prior to dose intake and at 1, 2, 4, 6 and 8 h after dose intake after 2 weeks’ inpatient treatment. According to the previously reported study (Kamp et al., 2017), the limited sampling strategy (predose and 2 h after dose intake) proven to have an accurate predication, comparable to intensive sampling (root mean squared error of 6.07%, *R* ^2^ of .98). Thus, in the validation cohort, limited sampling strategy were applied at predose, 2 and 6 h after dose intake after 2 weeks’ TB treatment. Plasma concentrations were determined using a validated high performance liquid chromatography tandem mass spectrometry (HPLC-MS/MS) method. Linezolid concentrations were measured using linezolid-d3 as the internal standard with m/z of 338.01→296.04. The analytical range for linezolid was .05–30 mg/L, with good linearity of *R* ^2^ ≥ 99.53%. The inaccuracy was within the range of 87.3%–108% for all concentrations and the imprecision values were less than 11.5% over the entire range of calibration standards.

### 2.3 Population pharmacokinetic modeling

To build the population PK model, data of 168 study participants from a previously published study were included (Zheng et al., 2021). The population PK model for linezolid was built using the nonlinear mixed-effects method (Phoenix NLME, version 8.0; Certara Inc., Princeto1n, United States). One- and two-compartment models with first-order eliminations were used to fit the data. The residual-error models included additive, proportional, and combined error models were tested. After building the structural model, covariates were added in a stepwise regression with forward inclusion (∆OFV>3.84, *p* <.05) and backward elimination (∆OFV>6.64, *p* <.01). The final model was evaluated and validated using visual predictive check (VPC) by simulating linezolid concentrations for 1,000 participants from the original data set and the final model. The population PK parameters in the validation cohort were calculated by Bayes Estimation based on the established population PK model.

### 2.4 Drug susceptibility testing

Sputum samples were collected at each visit and were sent to the prefectural TB reference laboratory for the microbiological examination. The BACTEC MGIT 960 system (Becton Dickinson, Franklin Lakes, NJ, United States) was used for bacterial culture of the *M. tb* isolates, phenotypic drug susceptibility testing and MIC determination for linezolid (Springer et al., 2009). All suspension from the growth in the plain MGIT medium were used within 3 days after found positive in MGIT incubator. The growth control tube containing 1:100 diluted bacterial suspension, was inoculated in the MGIT 960 instrument as well. The range of concentrations for MIC testing was .06–1 mg/L for linezolid. The MIC was defined as the lowest concentration of a drug that inhibited the bacterial growth. *M. tb* H37Rv (ATCC 27294) was used as the reference strain for the quality control.

### 2.5 Treatment outcome

The routine follow-up examinations and laboratory tests were performed monthly during the intensive phase, and every second month during the continuation phase of standardized MDR-TB treatment. Cure was defined as completed treatment and at least three consecutive, negative sputum cultures of *M. tb*, with at least 30 days in between sampling. A successful treatment outcome was defined as treatment completion or cure. An unsuccessful treatment outcome included treatment failure, all-cause mortality, and default during treatment or transfer out (World Health, 2013).

### 2.6 Identification and validation of PK/PD threshold of MDR-TB treatment outcome

The threshold was identified by relating the PK parameters to the treatment outcomes using boosted classification and regression tree analyses (CART). Boosted CART analysis was performed using Salford Predictive Miner System software (San Diego, CA, United States). Boosted CART analysis searched the PK parameters including peak serum concentration (C_max_), trough concentration (C_min_), AUC_0–24h_, AUC_0-24h_/MIC, C_max_/AUC_0–24h_ and the possible cutoff values to identify the best predictor for classifying between participants with and without the studied outcome (i.e., time to sputum culture conversion and successful treatment outcome) using Salford Predictive Miner System software (San Diego, CA, United States). The association between the boosted CART-derived threshold and treatment outcomes was validated by Poisson regression model with robust variance. COX proportional hazard regression model was used for evaluating the relationship between the boosted CART-derived threshold and time to sputum culture conversion. The clinical significance of the identified threshold was also validated by comparing to the previously reported threshold (119) derived from a hollow fiber infection model using the MGIT assay (Becton Dickinson, Franklin Lakes, NJ) (Srivastava et al., 2017).

### 2.7 Dose regimen evaluation

The Monte Carlo simulation was performed using Phoenix NLME (version 8.0; Certara Inc., Princeton, United States) as well. The characteristic data of a specifically simulated population (*n* = 1000) needed in the model were duplicated from original validation cohort to ensure its representativeness of the study population. The WHO-recommended dose (600 mg daily) (World Health, 2020), and the other previously proposed doses (300 mg, 900 mg, and 1200 mg daily) were evaluated by an analysis of the probability of target attainment (PTA) in the simulated population (Bolhuis et al., 2018). The PTA was derived by calculating the fraction of subjects who attained the PK/PD target or threshold at different MICs in BACTEC MGIT 960 system. The studied MICs included .06, .12, .25, .5, and 1 mg/L, where 1 mg/L of linezolid was referred to as the critical concentration in MGIT system in the Technical Report on critical concentrations for drug susceptibility testing (World Health, 2018). The dose was defined as sufficient at PTA values of ≥ 90%.

### 2.8 Statistical analysis

Baseline characteristics were summarized using descriptive statistics expressed as medians with interquartile ranges (IQR) for continuous variables and proportions for categorical variables. Chi-squared test analysis was performed for categorical variables, while one-way analysis of variance or Mann-Whitney *U* test were used for continuous variables. The main microbiological outcome was time to sputum culture conversion, defined as the time from treatment initiation to sustained sputum culture conversion. The time to sputum conversion was illustrated using the Kaplan-Meier method and difference between comparison groups were assessed using the log-rank test. Poisson regression model with robust variance was used to assess the correlation between the pharmacokinetic parameters and treatment outcomes. The multivariate COX proportional hazard regression model was used to verify the correlation between the CART-derived threshold and time to sputum culture conversion. The start time for the survival analysis was the first date of treatment. The endpoint of the observation was defined as the end of treatment in the survival analysis. As treatment outcome is influenced by multiple factors, we explored weight, BMI, tobacco use, alcohol use, diabetes mellitus type 2 status, albumin, cavity, baseline time to culture positivity and other factors that may be potential confounders based on the previous study (Madzgharashvili et al., 2021; Meregildo-Rodriguez et al., 2022). The association between linezolid drug exposure and treatment outcome were adjusted by the identified covariates based on the univariate analysis. A *p*-value of <.05 was considered statistically significant. IBM SPSS 20.0 (IBM Corp., Armonk, NY) was used to perform statistical description and COX proportional hazard regression model analysis.

## 3 Result

### 3.1 Population characteristics

The study included a total of 327 study participants. Data of 168 participants was used for the development cohort and data of 159 participants for the validation cohort. There was no significant difference between the two cohorts regarding the baseline characteristics (Table 1).

**TABLE 1:** Baseline demographic and clinical characteristics of participants in two studied cohorts.

Linezolid	Development cohort (n = 168)	Validation cohort (n = 159)	*P*-value^a^
	Median (IQR) or no. (%)	Median (IQR) or no. (%)	
Age, year	41 (33-45)	40 (29-54)	.47
Bodyweight, kg	53 (47-66)	59 (53-64)	.10
BMI	20 (18-23)	21 (18-22)	.95
Male	125 (74.4)	103 (64.8)	.08
Smoking	43 (25.6)	31 (19.5)	.24
Alcohol consumption	40 (23.8)	28 (17.6)	.12
Diabetes type 2	32 (19.0)	27 (17.0)	.49

Abbreviations: IQR, inter quartile range.

^a^ A Chi-square test were used to identify the differences between groups for categorical variables, while one-way analysis of variance or Mann-Whitney *U* test were used for continuous variables. The body mass index (BMI, kg/m^2^) was calculated through weight (kg) divided by square of height (m).

### 3.2 Drug susceptibility

**FIGURE 1:**
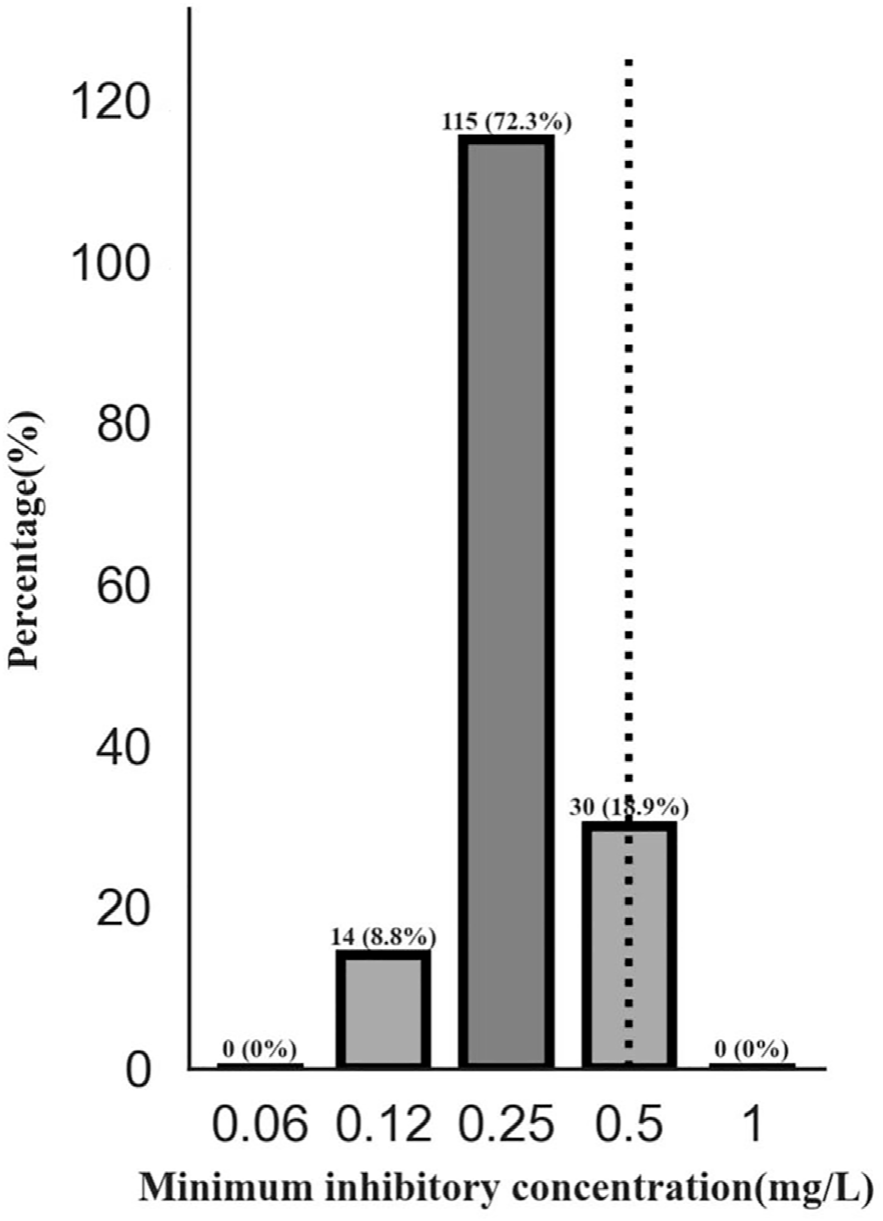
The distribution of minimum inhibitory concentration of linezolid for *Mycobacterium tuberculosis* isolates. Note: The BACTEC MGIT 960 system (Becton Dickinson, Franklin Lakes, NJ, United States of America) was used for bacterial culture, phenotype drug susceptibility testing and MIC values. The H37Rv (ATCC 27294) was used for reference to be inoculated with four batches of studied isolates as denoted. The MIC was defined as the lowest concentration of a drug that inhibited the bacterial growth. Abbreviations: MIC, minimum inhibitory concentration.

The median MIGT MICs of the clinical isolates were .25 (range .12–.5) mg/L for linezolid, with MIC ≤ .25 mg/L for the majority (81.1%) of the isolates. The H37Rv (ATCC 27294) as reference had a MIC of .5 mg/L, comparable to that in WHO report (World Health, 2018) (Figure 1). All strains were susceptible to linezolid before initiating the treatment.

### 3.3 Population pharmacokinetic modeling and parameter calculation

**FIGURE 2:**
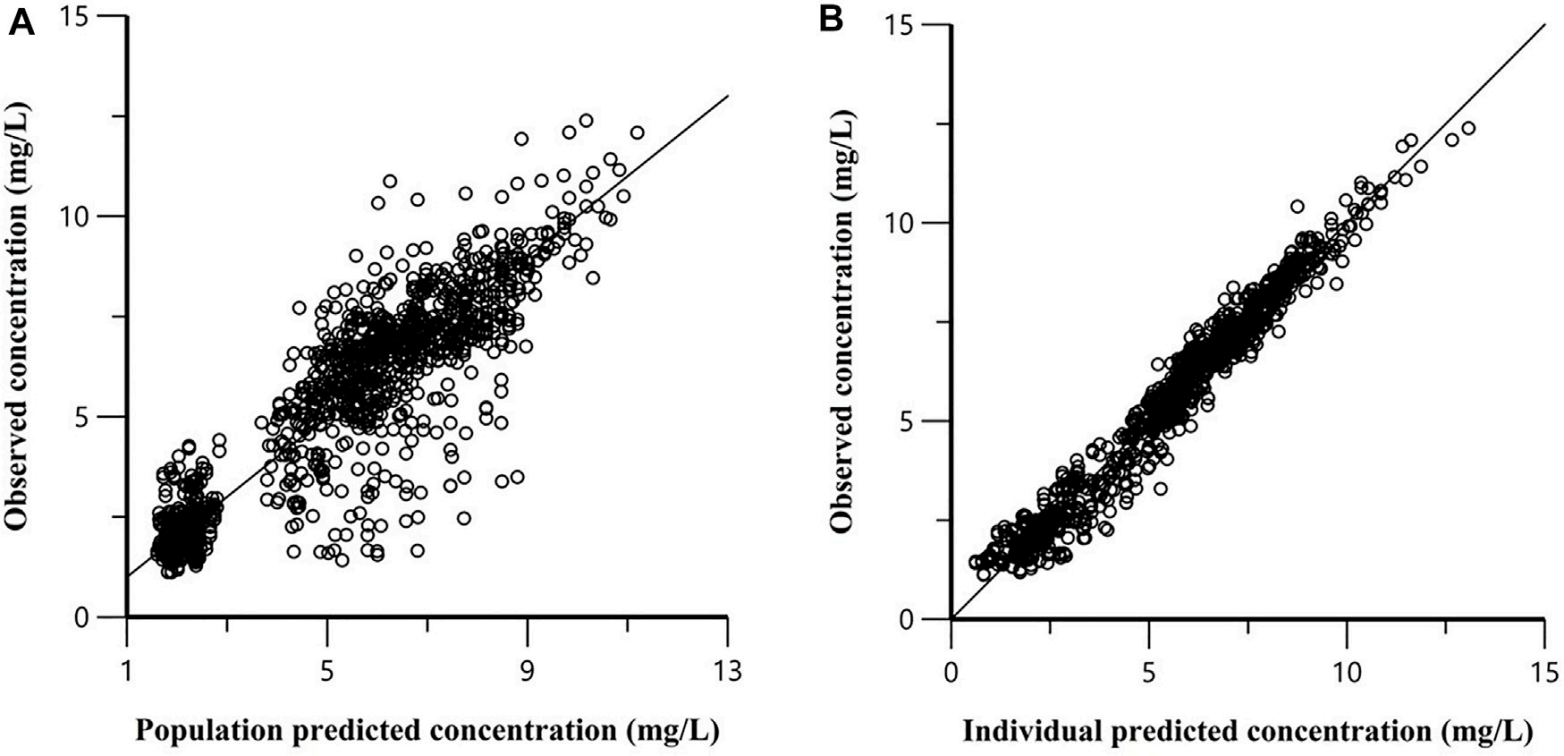
Goodness-of-fit plot for the final model. **(A)** Population predicted *versus* population observed concentrations; **(B)** Individual predicted *versus* individual observed concentrations.

**FIGURE 3:**
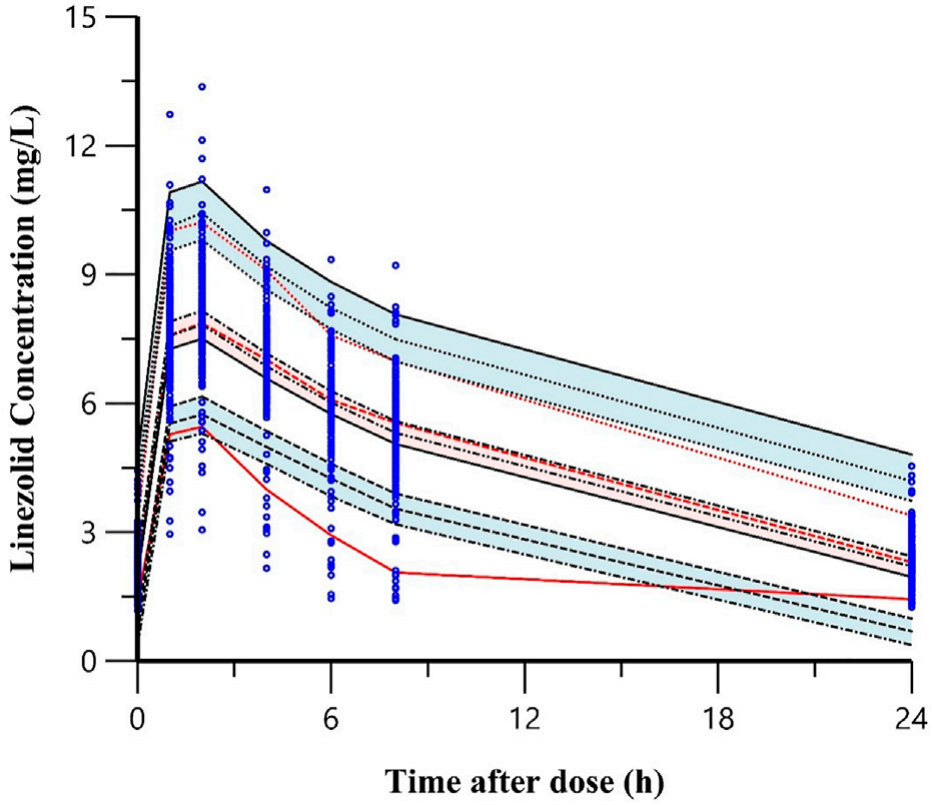
Visual predictive check plots of the final model for linezolid in the development cohort. Note: The top, middle, and bottom solid lines were the 95th,50th and 5th percentiles of the observed data, respectively. The shaded areas from top to bottom were the 95% confidence interval for the 95th, 50th, and 5th percentile of the simulated data. The dots were the observed concentrations.

The linezolid concentrations in 1008 plasma samples from 168 subjects were best described by a 2-compartment model with first-order absorption and elimination. An additive error model was used to describe the unexplained residual variability for linezolid. Apart from weight, diabetes type 2 independently influenced linezolid CL and V_d_ and the inclusion of diabetes type 2 resulted in a reduction of 38.49 points (*p* <.001) in OFV and explained 3.6% between-subject variability in CL and .6% between-subject variability in V_d_ (Table 2). The predicted linezolid concentrations reached an acceptable agreement with the observed concentrations, as shown in the goodness of fit and visual predictive check plots (Figure 2). The VPC for the entire data set demonstrated a good prediction of the model (Figure 3).

**TABLE 2:** Pharmacokinetic parameters of linezolid population pharmacokinetic model in development cohort.

	Linezolid	
	Mean	RSD (%)
Typical value of Population parameters		
Ka (/h)	2.0	11.1
CL (L/h)	5.6	1.6
V_d_(L)	35.8	2.0
Q (L/h)	.9	13.7
Vp(L)	58.6	13.2
Tlag(h)	.6	8.4
Variation of parameters between individuals		
CL (L/h)	21.6	14.6
Vd (%CV)	24.4	11.1
Tlag(%CV)	—	—
Covariate		
θ (Bodyweight)-CL	.75	—
θ (Bodyweight)-Vd	1.0	—
θ (Diabetes)-CL	.1	10.5
θ (Diabetes)-Vd	5.0	11.1
θ (Age)-CL	—	—
Additional residual (mg/L)	.5	4.2
Proportion of residual (mg/L)	—	—

Abbreviations: CL, clearance; CV, coefficient of variation; Ka, absorption rate constant; Vc, volume of central compartment distribution; Q, Inter-compartment clearance; Vp, volume of peripheral compartment; Tlag, lag time; V/F, apparent volume of distribution; RSD%, relative standard deviation.

Applying the population PK modeling, using the linezolid concentration measured in the samples from the validation cohort, the C_min_ was 2.0 (1.5–2.3) mg/L and the C_max_ was 16.2 (14.8–18.8) mg/L. The AUC_0–24h_ was 108.3 (82.6–119.1) mg h/L after an oral dose of 600 mg daily. The AUC_0–24h_/MIC values (median and IQR) were 428.3 (301.2–489.8). The C_max_/MIC values (median and IQR) were 64.0 (45.8–77.1) (Table 3).

**TABLE 3:** Pharmacokinetic parameters between groups with different treatment outcomes in validation cohort.

Pharmacokinetic parameters	Total	Successful treatment	Unsuccessful treatment	*P*-value^a^
	Median (IQR)	Median (IQR)	Median (IQR)	
C_max_ (mg/L)	16.2 (14.8–18.8)	16.4 (15.2–18.9)	13.4 (12.4–15.0)	<.01
C_min_(mg/L)	2.0 (1.5–2.3)	2.0 (1.6–2.3)	1.2 (.8, 1.3)	<.001
AUC_0-24h_ (mg h/L)	108.3 (82.6–119.1)	108.8 (87.1–120.6)	50.1 (48.0–56.4)	<.001
AUC_0-24h_/MIC	428.3 (301.2–489.8)	434.6 (326.1–491.8)	100.1 (96.0–112.8)	<.001
C_max_/MIC	64.0 (45.8–77.1)	64.7 (49.4–77.5)	26.8 (24.9–30.0)	<.001

Abbreviations: IQR, inter quartile range; 95% CI, 95% confidence interval; peak serum concentration (C_max_); trough concentration (C_min_); 0- to 24-h area under the concentration-time curve (AUC_0-24h_); minimum inhibitory concentrations (MIC).

^a^ Comparisons of C_max_, C_min_, AUC_0–24h_, AUC_0–24h_/MIC, C_max_/AUC_0-24h_ between successful treatment outcome group and unsuccessful treatment outcome groups were evaluated using one-way analysis of variance or Mann-Whitney *U* test.

### 3.4 Treatment management in validation cohort

During the treatment, 27 of the study participants experienced linezolid-induced toxicity presenting as cytopenia (14, 8.8%), peripheral neuropathy (11, 6.9%) and optic neuritis (9, 5.7%). Among 18 participants with the linezolid dose reduction or temporary interruption due to severe adverse events, all recovered and 17 participants were back to the standard dosage and one participant continued with a reduced dose of 300 mg daily until end of treatment.

At end of treatment, 149 (93.7%) succeeded in the treatment and median time of sputum conversion was 3 (1, 5) months. Except for sex and CXR-severity, there is no significant difference between the participants with and without successful treatment in terms of sociodemographic characteristic and baseline disease status (Table 4).

**TABLE 4:** Socio-demographic features and baseline disease status between groups with different treatment outcomes in validation cohort.

Variables	Successful treatment (n = 149) median (IQR) or no. (%)	Unsuccessful treatment (n = 10) median (IQR) or no. (%)	P Value^a^
Socio-demographic characteristics			
Age, year	40 (29.0, 53.5)	39 (30.3, 57.0)	.80
Height, m	1.70 (1.65, 1.77)	1.65 (1.62, 1.78)	.44
Bodyweight, kg	59 (53, 64)	59.5 (55.5, 69.0)	.24
BMI	20.1 ± 2.99	21.7 ± 3.29	.58
Sex, male	100 (67.1%)	3 (30%)	<.05
Baseline disease status			
Diabetes type 2	26 (17.4)	1 (10.0)	1.00
Albumin, g/L	42 (40.8, 52.0)	42 (40.8, 52.0)	.14
Clinical severity^b^	37 (24.8)	4 (40.0)	.28
CXR severity	22 (14.8)	4 (40.0)	<.05
Cavity	29 (19.5)	4 (40.0)	.22
Baseline time to culture positivity, day	13 (12.5, 15.0)	12 (10.0, 13.0)	.15

^a^ Comparisons of continuous variables between successful treatment outcome group and unsuccessful treatment outcome groups were evaluated using one-way analysis of variance or Mann-Whitney *U* test. Categorical variables was performed using the Chi-square or Fisher’s exact test.

^b^ Clinical severity was defined as the TB score ≥8 (Rudolf et al., 2013).

### 3.5 Identification and validation of clinical-significant thresholds

**FIGURE 4:**
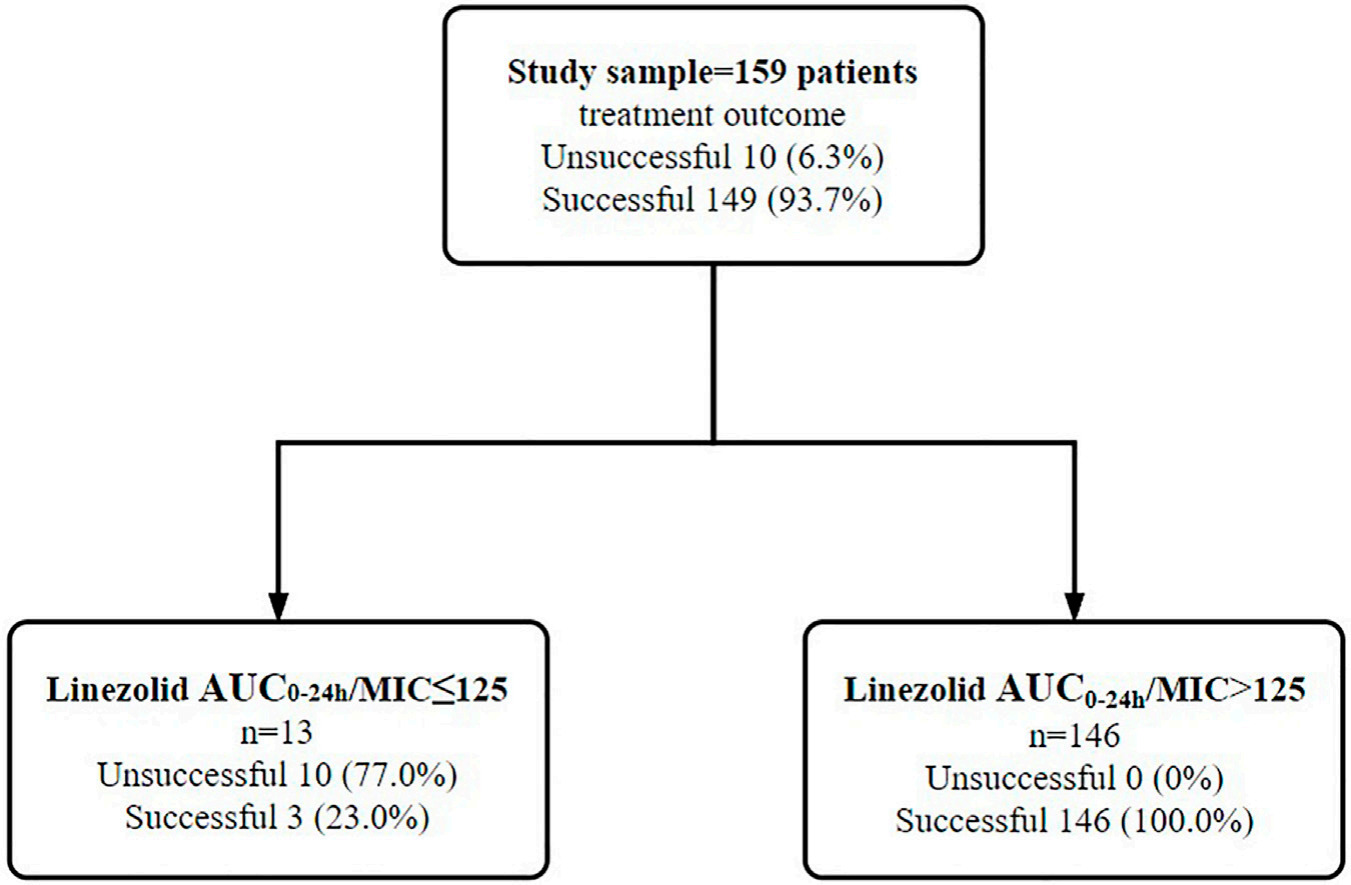
Identification of linezolid exposure/susceptibility threshold to differentiate the treatment outcome among 159 study participants in validation cohort. Note: AUC_0–24h_/MIC of linezolid were examined in the boosted classification and regression tree analyses. Abbreviations: MIC, minimum inhibitory concentration; AUC0-24 h, 0- to 24-h area under drug concentration-time curve.

**FIGURE 5:**
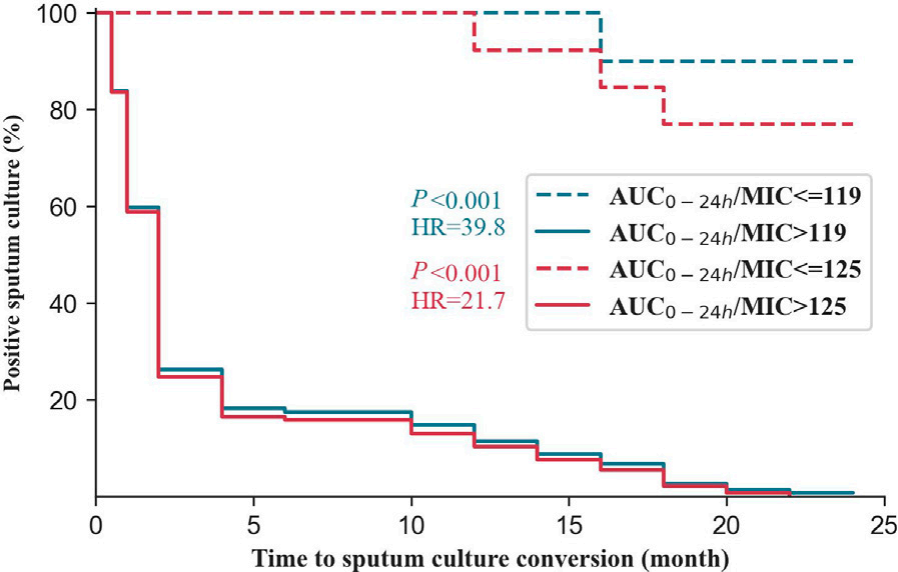
Time to culture conversion among the studied participants in validation cohort with multidrug-resistant tuberculosis grouped by the threshold in this study and previously reported target. Note: AUC_0–24h_/MIC of 125 was derive from boosted classification and regression tree analyses in the study. The previously reported target of AUC_0–24h_/MIC 119 (Srivastava et al., 2017)was identified using the MGIT assay (Becton Dickinson, Franklin Lakes, NJ) to identify MIC in hollow fiber infection model as the reference for comparison. Abbreviations: AUC0-24 h: 0- to 24-h area under the concentration-time curve; MIC: minimum inhibitory concentrations.

PK parameters including C_max_, C_min_, AUC_0-24h_, AUC_0-24h_/MIC and C_max_/AUC_0-24h_ were significantly different between groups of successful and unsuccessful treatment (Table 3). By relating PK/PD parameters to treatment outcome and time to sputum culture conversion, the CART-derived threshold of AUC_0–24h_/MIC (125) was identified (Figure 4). The proportion of study participants above the CART-derived threshold was 91.8% (146/159). The association between the CART-derived threshold and final treatment outcome was validated by Poisson regression model with robust variance (100% vs. 23.1%; adjusted RR, 4.3; 95%CI, 1.6–11.7). Also, the CART-derived threshold was significantly associated with time to sputum conversion (median time to sputum culture conversion between the group with AUC_0-24h_/MIC above and below 125: 2 vs. 24 months; adjusted HR, 21.7; 95%CI, 6.4–72.8) (Table 5). The association between the CART-derived threshold and final treatment outcome/time to sputum culture conversion was well comparable to the previously reported target of 119 (Srivastava et al., 2017) (Figure 5).

**TABLE 5:** Validation of CART-derived threshold in relation to the final treatment outcome and time to sputum culture conversion in the validation cohort.

Thresholds of AUC_0-24h_/MIC	Treatment outcome			Time to sputum culture conversion		
	Successful (%)	RR	aRR	Median (IQR) time to culture conversion (month)	HR (95%CI)	Adjusted HR (95%CI)^b^
≤125	3 (23.1)	1	1	24 (24, 24)	1	1
>125	146 (100)	4.3 (1.6, 11.7)	4.3 (1.6, 11.7)	2 (1, 4)	20.4 (6.3, 66.6)	21.7 (6.4, 72.8)
≤119^a^	0 (0)	1	1	24 (24, 24)	1	1
>119^a^	149 (100)	9.9 (1.6, 63.8)	9.9 (1.6, 63.8)	2 (1, 4)	43.5 (6.0, 316.6)	39.8 (5.4, 292.5)

Abbreviations: AUC_0-24h_: the 0- to 24-h area under drug concentration-time curve; MIC: minimum inhibitory concentration in BACTEC 960 MGIT; RR, relative risk; HR, hazard ratio. CART: classification and regression tree analyses.

^a^ The previously reported target of AUC_0–24h_/MIC 119 (Srivastava et al., 2017) was identified using the MGIT assay (Becton Dickinson, Franklin Lakes, NJ) to identify MIC in hollow fiber infection model as the reference for comparison.

^b^ The association between the boosted classification and regression tree analyses (CART)-derived threshold and treatment outcome was validated by Poisson regression model with robust variance. COX proportional hazard regression model was used for evaluating the relationship between the boosted CART-derived threshold and time to sputum culture conversion. Adjusted HR was calculated according to current area, age, sex; CXR severity.

### 3.6 Dose regimen evaluation

**FIGURE 6:**
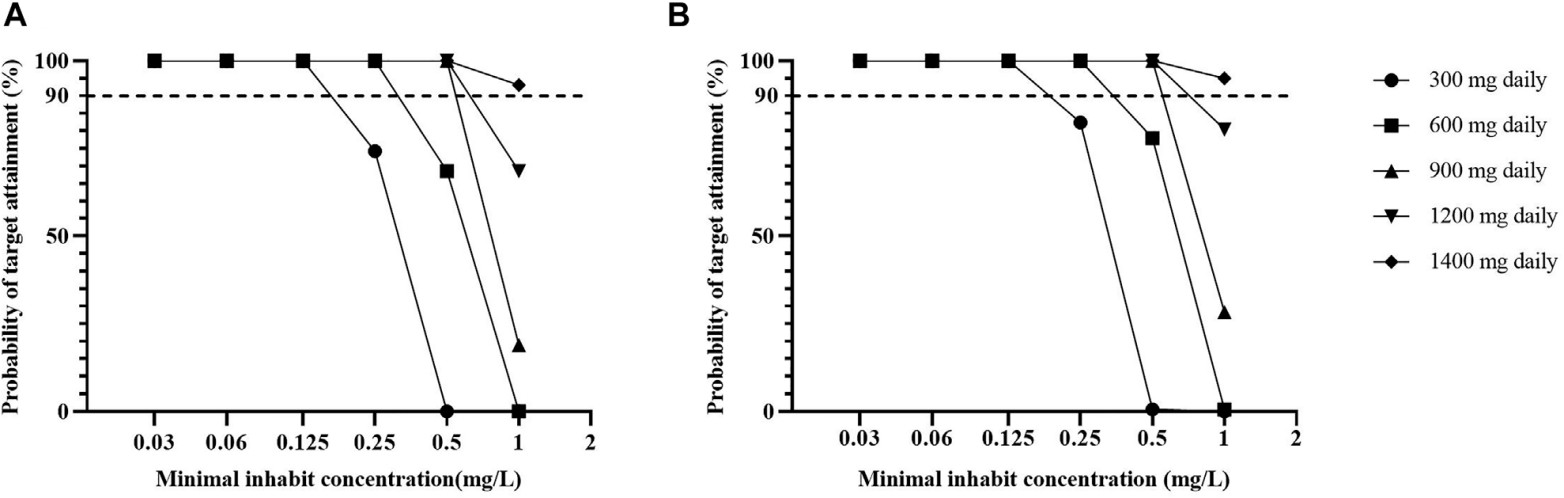
The probability of target attainment among the simulating population against varying MGIT minimal inhibitory concentration values for linezolid respectively based on the AUC_0–24h_/MIC of 125 in this study **(A)** and the 119 previously reported **(B)**. Note: The previously reported target of AUC_0–24h_/MIC 119 (Srivastava et al., 2017) was identified using the MGIT assay (Becton Dickinson, Franklin Lakes, NJ) to identify MIC in hollow fiber infection model as the reference for comparison. Abbreviations: PTA, probability of target attainment; AUC_0-24h_: 0- to 24-h area under the concentration-time curve; MIC, minimum inhibitory concentration.

Based on the CART-derived threshold, at a standard linezolid dose of 600 mg daily, PTA was simulated to achieve 100% at MGIT MICs of ≤ .25 mg/L, while at 300 mg daily, commonly used when adverse events happen, PTA attain above 90% at MICs ≤ .125 mg/L and was 74.2% at MIC of .25 mg/L. Comparably, a dose of 900 mg daily had PTA exceeding 90% (100%) at an MIC of .5 mg/L covering all isolates in our study. At the critical concentration of 1 mg/L in MGIT which was not found for any isolate in our study, 1200 mg daily failed to achieve a PTA of ≥ 90% (68.6%) at the AUC_0-24h_/MIC ratio of 125. Only dose up to 1400 mg had PTA exceeded 90% (93.1%) at MIC of 1 mg/L (Figure 6).

## 4 Discussion

Our results provide valuable insight into population PK and its association with time to sputum culture conversion and treatment outcome as well as the probability of target attainment with current WHO recommended regimen in participants with MDR-TB in China. A linezolid PK/PD threshold of AUC/MIC > 125, using MGIT MICs, was associated with successful treatment outcome and may be used for TDM.

We found that two-compartment model with an additive error model best fitted the pharmacokinetic profiles of linezolid. Previous studies reported a one-compartment model (Sotgiu et al., 2012; Kamp et al., 2017; Lopez et al., 2019; Alghamdi et al., 2020) to be adequate to describe the pharmacokinetic profile of linezolid while our study found that a two-compartment described data better compared to a one-compartment model as demonstrated by an OFV decrease of 18. This can be explained by smaller sample sizes or collection of fewer blood samples in the elimination phase of the drug. Diabetes type 2 was found to be a major covariate explaining inter-individual residual variability of clearance and distribution volume for linezolid. Participants with diabetes type 2 commonly take the risk of developing diabetic gastroparesis, thus may affect the absorption of drugs. In previous studies (Singla et al., 2006), TB participants with diabetes type 2 were found to have higher probability of suboptimal drug exposure, which was explained by malabsorption due to diabetic enteropathy or by increased BMI (Chang et al., 2011; Mtabho et al., 2019). The VPC results indicated that the developed model was precise and could be used for simulation purposes. Thus, we established a population PK modeling suitable for TDM of linezolid during the MDR-TB treatment.

We identified an association between linezolid exposure and clinical treatment outcome of MDR-TB. Linezolid is a concentration-dependent drug and high drug exposure contributes to treatment efficacy, albeit limited by adverse events (Deshpande et al., 2016). As an oxazolidinone with potent activity against *M. tb*, linezolid suppresses oxidative-phosphorylation protein complexes 1, 3, 4, and 5 and ATP production levels in a clearly exposure-dependent manner for the once-daily (q24 h) regimens (Brown et al., 2015). This present study observed 27 of participants had linezolid-induced toxicity, some of which are thought to be associated with mitochondrial disturbance. However, after dose reduction or interruption, all recovered. Meanwhile, we did not find the impact of mitochondrial toxicity on the treatment outcome, probably due to the healthier participant included in the present study compared to the previously published studies (Brown et al., 2015; Song et al., 2015). In the present study, AUC/MIC was identified to be related to the treatment outcome, which is also demonstrated by previous studies (Alffenaar et al., 2011; Sotgiu et al., 2015).

An AUC_0–24h_/MIC > 125, applying MGIT MICs was identified by boosted CART as primary node to define the successful treatment outcome of longer regimen in MDR-TB participants among our study population. This threshold was also observed to be strongly associated with the treatment outcome and time to sputum culture conversion, which is supported by the previously reported target for optimal bactericidal activity of an AUC_0–24h_/MIC >119 (Srivastava et al., 2017). By confirming the clinical significance, this threshold may be applied for dose adjustments in a randomized controlled trial investigating TDM-derived doses of linezolid vs. standard dose of linezolid for MDR-TB treatment before applying it in the routine medical practice.

In the present study, currently recommended dose of linezolid was observed to be effective at MIC ≤ .25 mg/L which covered the majority of the isolates (81.1%). Based on the identified threshold, 600 mg would have a PTA exceeding 90% at MIC ≤ .25 mg/L in MGIT. The clinical efficacy of 600 mg linezolid for susceptible isolates (MIC ≤ .25 mg/L) in Middlebrook 7H10 agar plates is also reported in previous studies (Heinrichs et al., 2019; Alghamdi et al., 2020). In our exploratory analysis, we found PTAs below 90% for 600 mg of linezolid at MICs of .5 and in particular 1 mg/L. Of note, we found no isolate at a MIC of 1 mg/L in our study. Furthermore, it should be noted that if individual TDM is considered at the suggested targets, the technical MIC variability of ± one MIC dilution must be considered since this variability may affect the individual PK/PD value significantly. When considering higher dosing than 600 mg of linezolid daily, it should be noted that the administration of 1200 mg daily in the Nix-TB study was reported to achieve as high as 90% favorable outcome, although with alarming high rates of severe adverse events in the study participants (81% peripheral neuropathy and 48% myelosuppression) (Conradie et al., 2020). Therefore, clinically validated PK/PD thresholds and individual-based dose-guidance by TDM are important tools for dose optimization to avoid adverse events while ensuring treatment efficacy.

The strength of our study is that we developed population PK models for linezolid based on a relatively large number of MDR-TB participants with a standardized MDR-TB treatment regimen in China, where similar studies have not been reported. Additionally, the PK/PD threshold in this study was identified by treatment outcome of a large clinical cohort population, which can provide valuable and clinically-relevant support for the dose adjustment of linezolid using TDM. Another strength is that this study evaluated the validation of current WHO-recommended dose for linezolid using simulations and demonstrated a high target attainment (≥ 90%). Furthermore, the study provided the important example to suggest that, to use the limited sampling for monitoring in daily practice with population pharmacokinetic model, would support AUC guided dosing. Regarding linezolid monitoring in a clinical setting, current recommendations include a trough concentration <2.5 mg/L (Wasserman et al., 2022) or < 2 mg/L (Song et al., 2015) relevant to linezolid-relevant adverse events as well as drug exposure/susceptibility thresholds predictive of favorable treatment outcome (e.g., 125 in present study). Their clinical significances for the dosing adjustment will require the validation from the randomized clinical trials.

This study is subject to some limitations. Firstly, as the study participants required to be healthy enough to receive the whole course of treatment, none die or experienced unmanageable adverse events, which may restrict the representativeness of the study findings to some degree. As repeated DST testing of consecutive *M. tb* cultures was not performed, we were unable to monitor the potential development of linezolid resistance during treatment. However, since a high proportion of participants had sputum cultured converted at 6 months’ treatment (77.4%), and that resistance emergence against linezolid is extremely rarely reported in the literature (Lee et al., 2017; Wasserman et al., 2019), we regard the risk for undetected acquired drug resistance to linezolid very low. Meanwhile, during the treatment, we retrieved the data of treatment prognostics from the medical records and we are unable to find the possible impact of treatment-related factors (e.g., acquired resistance to linezolid) on the treatment outcome. Additionally, the treatment outcome may be influenced by baseline disease status, the association between linezolid and treatment outcome has been adjusted for age, sex, area, and CXR severity. The treatment outcome of MDR-TB is the result of the complete treatment regimen. Hence, the observed threshold concentrations associated with successful treatment outcome need to be viewed in context of the provided treatment and setting. Given the comparable results in the development and the validation cohort, we feel confident that results can likely be extrapolated to other settings in China. However, the PK/PD threshold should be used with caution in settings outside China. Additionally, the MIC distribution has substantial influence on the PK/PD analyses and MIC determinations show technical variability within and between different methods and laboratories. Thus, some our target may need further validation before generalized to other populations and MIC methods.

## 5 Conclusion

We reported an AUC_0-24h_/MIC threshold of 125 associated with clinical outcomes in the MDR-TB participant in China, which may serve as a target for dose adjustment of linezolid to improve treatment outcome. Linezolid exposures associated with the WHO-recommended dose (600 mg daily) was sufficient in our setting for the majority of MDR-TB isolates and sufficient for all isolates with MGIT MIC≤.25 mg/L.

## Data Availability

The original contributions presented in the study are included in the article, further inquiries can be directed to the corresponding author.

## References

[B1] Ahmad N. Ahuja S. D. Akkerman O. W. Alffenaar J. C. Anderson L. F. Baghaei P. (2018). Treatment correlates of successful outcomes in pulmonary multidrug-resistant tuberculosis: An individual patient data meta-analysis. Lancet 392 (10150), 821–834. 10.1016/s0140-6736(18)31644-1 30215381 PMC6463280

[B2] Alffenaar J. W. C. Van Der Laan T. Simons S. Van Der Werf T. S. Van De Kasteele P. J. De Neeling H. (2011). Susceptibility of clinical *Mycobacterium tuberculosis* isolates to a potentially less toxic derivate of linezolid, PNU-100480. Antimicrob. Agents Chemother. 55 (3), 1287–1289. 10.1128/AAC.01297-10 21199931 PMC3067097

[B3] Alghamdi W. A. Al-Shaer M. H. An G. Alsultan A. Kipiani M. Barbakadze K. (2020). Population pharmacokinetics of linezolid in tuberculosis patients: Dosing regimen simulation and target attainment analysis. Antimicrob. Agents Chemother. 64 (10), e01174. 10.1128/aac.01174-20 32778547 PMC7508612

[B4] Bolhuis M. S. Akkerman O. W. Sturkenboom M. G. G. Ghimire S. Srivastava S. Gumbo T. (2018). Linezolid-based regimens for multidrug-resistant tuberculosis (tb): A systematic review to establish or revise the current recommended dose for tb treatment. Clin. Infect. Dis. 67, S327–s335. 10.1093/cid/ciy625 30496467

[B5] Brown A. N. Drusano G. L. Adams J. R. Rodriquez J. L. Jambunathan K. Baluya D. L. (2015). Preclinical evaluations to identify optimal linezolid regimens for tuberculosis therapy. mBio 6 (6), e01741–e01715. 10.1128/mBio.01741-15 26530386 PMC4631805

[B6] Chang J. T. Dou H. Y. Yen C. L. Wu Y. H. Huang R. M. Lin H. J. (2011). Effect of type 2 diabetes mellitus on the clinical severity and treatment outcome in patients with pulmonary tuberculosis: A potential role in the emergence of multidrug-resistance. J. Formos. Med. Assoc. 110 (6), 372–381. 10.1016/s0929-6646(11)60055-7 21741005

[B7] Conradie F. Diacon A. H. Ngubane N. Howell P. Everitt D. Crook A. M. (2020). Treatment of highly drug-resistant pulmonary tuberculosis. N. Engl. J. Med. 382 (10), 893–902. 10.1056/NEJMoa1901814 32130813 PMC6955640

[B8] Deshpande D. Srivastava S. Nuermberger E. Pasipanodya J. G. Swaminathan S. Gumbo T. (2016). Concentration-dependent synergy and antagonism of linezolid and moxifloxacin in the treatment of childhood tuberculosis: The dynamic duo. Clin. Infect. Dis. 63, S88–s94. 10.1093/cid/ciw473 27742639 PMC5064154

[B9] Heinrichs M. T. Drusano G. L. Brown D. L. Maynard M. S. Sy S. K. B. Rand K. H. (2019). Dose optimization of moxifloxacin and linezolid against tuberculosis using mathematical modeling and simulation. Int. J. Antimicrob. Agents 53 (3), 275–283. 10.1016/j.ijantimicag.2018.10.012 30385322

[B10] Kamp J. Bolhuis M. S. Tiberi S. Akkerman O. W. Centis R. de Lange W. C. (2017). Simple strategy to assess linezolid exposure in patients with multi-drug-resistant and extensively-drug-resistant tuberculosis. Int. J. Antimicrob. Agents 49 (6), 688–694. 10.1016/j.ijantimicag.2017.01.017 28389352

[B11] Lee J. Y. Kim D. K. Lee J.-K. Yoon H. I. Jeong I. Heo E. (2017). Substitution of ethambutol with linezolid during the intensive phase of treatment of pulmonary tuberculosis: Study protocol for a prospective, multicenter, randomized, open-label, phase II trial. Trials 18 (1), 68. 10.1186/s13063-017-1811-0 28193240 PMC5307889

[B12] Lopez B. Siqueira de Oliveira R. Pinhata J. M. W. Chimara E. Pacheco Ascencio E. Puyén Guerra Z. M. (2019). Bedaquiline and linezolid MIC distributions and epidemiological cut-off values for *Mycobacterium tuberculosis* in the Latin American region. J. Antimicrob. Chemother. 74 (2), 373–379. 10.1093/jac/dky414 30358851

[B13] Madzgharashvili T. Salindri A. D. Magee M. J. Tukvadze N. Avaliani Z. Blumberg H. M. (2021). Treatment outcomes among pediatric patients with highly drug-resistant tuberculosis: The role of new and repurposed second-line tuberculosis drugs. J. Pediatr. Infect. Dis. Soc. 10 (4), 457–467. 10.1093/jpids/piaa139 PMC8087132 33347564

[B14] Meregildo-Rodriguez E. D. Asmat-Rubio M. G. Zavaleta-Alaya P. Vásquez-Tirado G. A. (2022). Effect of oral antidiabetic drugs on tuberculosis risk and treatment outcomes: Systematic review and meta-analysis. Trop. Med. Infect. Dis. 7 (11), 343. 10.3390/tropicalmed7110343 36355885 PMC9694577

[B15] Mtabho C. M. Semvua H. H. van den Boogaard J. Irongo C. F. Boeree M. J. Colbers A. (2019). Effect of diabetes mellitus on TB drug concentrations in Tanzanian patients. J. Antimicrob. Chemother. 74 (12), 3537–3545. 10.1093/jac/dkz368 31651031 PMC7183353

[B16] Padayatchi N. Bionghi N. Osman F. Naidu N. Ndjeka N. Master I. (2020). Treatment outcomes in patients with drug-resistant TB-HIV co-infection treated with bedaquiline and linezolid. Int. J. Tuberc. Lung Dis. 24 (10), 1024–1031. 10.5588/ijtld.20.0048 33126934 PMC8141513

[B17] Pasipanodya J. G. McIlleron H. Burger A. Wash P. A. Smith P. Gumbo T. (2013). Serum drug concentrations predictive of pulmonary tuberculosis outcomes. J. Infect. Dis. 208 (9), 1464–1473. 10.1093/infdis/jit352 23901086 PMC3789573

[B18] Rudolf F. Lemvik G. Abate E. Verkuilen J. Schön T. Gomes V. F. (2013). TBscore II: Refining and validating a simple clinical score for treatment monitoring of patients with pulmonary tuberculosis. Scand. J. Infect. Dis. 45 (11), 825–836. 10.3109/00365548.2013.826876 24041274

[B19] Singh B. Cocker D. Ryan H. Sloan D. J. (2019). Linezolid for drug-resistant pulmonary tuberculosis. Cochrane Database Syst. Rev. 3 (3), Cd012836. 10.1002/14651858.CD012836.pub2 30893466 PMC6426281

[B20] Singla R. Khan N. Al-Sharif N. Ai-Sayegh M. O. Shaikh M. A. Osman M. M. (2006). Influence of diabetes on manifestations and treatment outcome of pulmonary TB patients. Int. J. Tuberc. Lung Dis. 10 (1), 74–79. 16466041

[B21] Song T. Lee M. Jeon H. S. Park Y. Dodd L. E. Dartois V. (2015). Linezolid Trough concentrations correlate with mitochondrial toxicity-related adverse events in the treatment of chronic extensively drug-resistant tuberculosis. EBioMedicine 2 (11), 1627–1633. 10.1016/j.ebiom.2015.09.051 26870788 PMC4740314

[B22] Sotgiu G. Centis R. D'Ambrosio L. Alffenaar J. W. Anger H. A. Caminero J. A. (2012). Efficacy, safety and tolerability of linezolid containing regimens in treating MDR-TB and XDR-TB: Systematic review and meta-analysis. Eur. Respir. J. 40 (6), 1430–1442. 10.1183/09031936.00022912 22496332

[B23] Sotgiu G. Centis R. D'Ambrosio L. Castiglia P. Migliori G. B. (2015). Low minimal inhibitory concentrations of linezolid against multidrug-resistant tuberculosis strains. Eur. Respir. J. 45 (1), 287–289. 10.1183/09031936.00135014 25552740

[B24] Springer B. Lucke K. Calligaris-Maibach R. Ritter C. Böttger E. C. (2009). Quantitative drug susceptibility testing of *Mycobacterium tuberculosis* by use of MGIT 960 and EpiCenter instrumentation. J. Clin. Microbiol. 47 (6), 1773–1780. 10.1128/jcm.02501-08 19339475 PMC2691107

[B25] Srivastava S. Magombedze G. Koeuth T. Sherman C. Pasipanodya J. G. Raj P. (2017). Linezolid dose that maximizes sterilizing effect while minimizing toxicity and resistance emergence for tuberculosis. Antimicrob. Agents Chemother. 61 (8), e00751. 10.1128/aac.00751-17 28584143 PMC5527615

[B26] Sturkenboom M. G. G. Märtson A. G. Svensson E. M. Sloan D. J. Dooley K. E. van den Elsen S. H. J. (2021). Population pharmacokinetics and bayesian dose adjustment to advance TDM of anti-TB drugs. Clin. Pharmacokinet. 60 (6), 685–710. 10.1007/s40262-021-00997-0 33674941 PMC7935699

[B27] Swaminathan S. Pasipanodya J. G. Ramachandran G. Hemanth Kumar A. K. Srivastava S. Deshpande D. (2016). Drug concentration thresholds predictive of therapy failure and death in children with tuberculosis: Bread crumb trails in random forests. Clin. Infect. Dis. 63, S63–s74. 10.1093/cid/ciw471 27742636 PMC5064152

[B28] Wasserman S. Brust J. C. M. Abdelwahab M. T. Little F. Denti P. Wiesner L. (2022). Linezolid toxicity in patients with drug-resistant tuberculosis: A prospective cohort study. J. Antimicrob. Chemother. 77 (4), 1146–1154. 10.1093/jac/dkac019 35134182 PMC7612559

[B29] Wasserman S. Denti P. Brust J. C. M. Abdelwahab M. Hlungulu S. Wiesner L. (2019). Linezolid pharmacokinetics in South African patients with drug-resistant tuberculosis and a high prevalence of HIV coinfection. Antimicrob. Agents Chemother. 63 (3), e02164. 10.1128/aac.02164-18 30617089 PMC6395899

[B30] Wasserman S. Meintjes G. Maartens G. (2016). Linezolid in the treatment of drug-resistant tuberculosis: The challenge of its narrow therapeutic index. Expert Rev. Anti Infect. Ther. 14 (10), 901–915. 10.1080/14787210.2016.1225498 27532292

[B31] World Health O. (2013). Definitions and reporting framework for tuberculosis – 2013 revision: Updated december 2014 and january 2020. Geneva: World Health Organization.

[B32] World Health O. (2018). Technical report on critical concentrations for drug susceptibility testing of medicines used in the treatment of drug-resistant tuberculosis. Geneva: World Health Organization.

[B33] World Health O. (2020). WHO consolidated guidelines on tuberculosis: Module 4: Treatment - drug-resistant tuberculosis treatment. Geneva: World Health Organization. 32603040

[B34] Zheng X. Davies Forsman L. Bao Z. Xie Y. Ning Z. Schön T. (2021). Drug exposure and susceptibility of second-line drugs correlate with treatment response in patients with multidrug-resistant tuberculosis: A multi-centre prospective cohort study in China. Eur. Respir. J. 59, 2101925. 10.1183/13993003.01925-2021 PMC8943270 34737224

